# Association of homoarginine with arginine and disease severity in COVID-19 patients

**DOI:** 10.1007/s00726-025-03453-6

**Published:** 2025-05-07

**Authors:** Zhiling Zhao, Ting-Ting Wei, Wan-Xue Zhang, Shan-Shan Zhang, Rui Wu, Fei Li, Han Yang, Qiang Zhang, Jingjing Xi, Yiguo Zhou, Tiehua Wang, Juan Du, Qing-Bin Lu, Qinggang Ge

**Affiliations:** 1 Department of Intensive Care Medicine, Peking University Third Hospital, 49 North Garden Rd., Haidian District, Beijing, 100191 China; 2 Department of Laboratorial Science and Technology and Vaccine Research Center, School of Public Health, Peking University, Beijing, China; 3 Center for Infectious Disease and Policy Research and Global Health and Infectious Diseases Group, Peking University, Beijing, China; 4 Department of Epidemiology and Biostatistics, School of Public Health, Peking University, Beijing, China; 5 Pulmonary and Critical Care Medicine, Peking University Third Hospital, Beijing, China; 6 Department of General Surgery, Peking University Third Hospital, Beijing, China; 7 Department of Health Policy and Management, School of Public Health, Peking University, Beijing, China; 8 Key Laboratory of Epidemiology of Major Diseases (Peking University), Ministry of Education, Beijing, China; 9 Center for Infectious Disease and Policy Research and Department of Laboratorial of Science and Technology, School of Public Health, Peking University, No 38 Xue‐Yuan Rd, Haidian District, Beijing, 100191 China

**Keywords:** Homoarginine, Arginine, Cytokine, COVID-19, Disease severity

## Abstract

This study explored the relationship between the concentrations of homoarginine and arginine and between homoarginine concentration and laboratory parameters in coronavirus disease 2019 (COVID-19) patients with different severity to demonstrate the role of homoarginine in the progress of COVID-19. The laboratory-confirmed COVID-19 patients were included from Peking University Third Hospital during December 2022 to January 2023. Serum, urine, and stool samples were collected from the patients and detected by liquid chromatography-mass spectrometry. Totally 46 patients were recruited, including 18 in the mild group, 19 in the severe group, and 9 fatal. The concentration of homoarginine was positively correlated with the concentration of arginine in serum (*r* = 0.50), urine (*r* = 0.55), and stool samples (*r* = 0.39), respectively (all P < 0.001). The serum concentration and urine concentration of homoarginine were lower in severe patients than in mild patients (both P < 0.05). 13 indicators reflecting immunity and coagulation, including but not limited to T cell, white blood cell, natural killer cell, interleukin 6 (IL-6), and IL-8, had statistically significant correlations with both disease severity and the homoarginine concentration. Patients with hypertension were significantly associated with the decreased serum homoarginine (odds ratio 10.905, 95% confidence interval 1.454 − 137.144). Our results suggest that the homoarginine plays a role in the progress of COVID-19, which may be achieved by influencing arginine metabolism.

**Supplementary Information:**

The online version contains supplementary material available at 10.1007/s00726-025-03453-6.

## Introduction

The coronavirus disease 2019 (COVID-19) caused by severe acute respiratory syndrome coronavirus 2 (SARS-CoV-2) has resulted in millions of confirmed cases and deaths worldwide (Shanthanna et al. 2022). To identify diagnostic and prognostic biomarkers, numerous studies have evaluated the effect of SARS-CoV-2 infection and COVID-19 severity on metabolites in plasma, urine, and stool samples (Bourgin et al. 2023).

As one of the important metabolites in the SARS-CoV-2 infection, arginine is considered to play a vital role in COVID-19 (Barberis et al. 2020; Danlos et al. 2021; Rees et al. 2021), mainly through its effects on nitric oxide (NO) production and immune function (Rees et al. 2021). Arginine promotes NO production, which has been proven to be a signaling molecule involved in numerous processes, including antiviral responses, oxidized phospholipids generation, and programmed cell death (Lisi et al. 2021). In addition, a large part of a normal immune function, including T cell and macrophage function, depends on arginine levels (Adebayo et al. 2021). The reduced availability of arginine was reported to be connected with decreased proliferation of T cells and diminished responses in T cell-mediated memory (Zhu et al. 2014) and the administration of arginine improved the host immune response (Tepaske et al. 2001). The lower concentration of plasmatic arginine was observed in COVID-19 patients compared with healthy controls (Rees et al. 2021) and in severe COVID-19 patients compared with moderate patients (Sacchi et al. 2021). However, arginine is substantially eliminated by arginases after oral administration, which requires relatively high oral doses to increase the plasma concentration of arginine (Morris 2004).

Homoarginine is an endogenous and nonproteinogenic amino acid and structurally related to arginine, and has potential physiological functions in humans as well (Atzler et al. 2015). On the one hand, homoarginine is generated from arginine and glycine by arginine: glycine amidinotransferase (AGAT) (Tsikas and Wu 2015). On the other hand, in spite of that the binding affinity (K_m_) of homoarginine was 10–20 times lower than that of arginine, homoarginine is a weak substrate for nitric oxide synthase (NOS) and thus has been considered a biomarker of cardiovascular disease (Atzler et al. 2015; Moali et al. 1998). Although it is controversial whether homoarginine is a substrate or an inhibitor of arginase, it can be hypothesized that the concentration of homoarginine theoretically has an effect on arginase level and even arginine concentration (Reczkowski and Ash 1994; Tommasi et al. 2018). Therefore, the relationship between the concentrations of homoarginine and arginine in humans remains unclear. Studies about the association between homoarginine concentration and disease severity in COVID-19 patients are limited. The study by Adnan et al. (Haşimi et al. 2023) determined the serum concentrations of arginine and homoarginine in 86 COVID-19 patients and 21 controls and showed no statistically significant associations with disease severity. However, the patients spanned a wide range of ages in the different groups. According to previous studies, the high arginine concentration are negatively correlated with the increase of age (Atzler et al. 2014). Therefore, more studies need to explore and validate the relationship between homoarginine and disease severity.

This study aimed to explore the effects of homoarginine on the progress of the COVID-19, especially on the concentrations of arginine and the association with clinical laboratory parameters in COVID-19 patients.

## Materials and methods

### Study design

The laboratory-confirmed COVID-19 patients aged ≥ 18 years old whose imaging shows the characteristic manifestations of COVID-19 pneumonia from December 2022 to January 2023 were included. Patients with lung tumor, bronchiectasis, interstitial lung disease, tuberculosis, pulmonary embolism, hepatitis, hyperthyroidism, hyperuricemia and those who had the history of lung surgery were excluded. The laboratory-confirmation of SARS-CoV-2 infection and severity of COVID-19 were confirmed according to Diagnosis and Treatment of Novel Coronavirus Pneumonia (Version 9) released by National Health Commission (Diagnosis and Treatment of Novel Coronavirus Pneumonia (Version 9). 2022). According to the outcomes, the severe COVID-19 patients were divided into survival and fatal groups. Serum samples, urine samples, and stool samples were collected from all COVID-19 patients, while samples were collected again in patients who survive severe infection before being discharged from the hospital. Patients were discharged from the hospital with normal blood tests, undetectable SARS-CoV-2 genome, and no obvious clinical symptoms.

The study protocol was approved by the Human Ethics Committee of Peking University Third Hospital and Informed consent was obtained from all the participants.

### Information and samples collection

Demographic data and clinical manifestations were collected from medical records. Demographic data consists of age, sex, body mass index (BMI), smoking status, drinking status, and underlying diseases. Clinical manifestations consisted of fever, the highest body temperature, cough, expectoration, dyspnea, polypnea, and wheezing. Serum samples, urine samples, and stool samples were collected by skilled nurses.

### Laboratory tests

The detection of lymphocyte subsets used Fluorescent Monoclonal Antibody Kits (Beijing Tongshengshidai Biotechnology Co., Ltd, Beijing, China, Cat. no.: Luqing Equipment 20,180,087). The cytokine detection reagent was provided by Qingdao Raisecare Biotechnology Co., Ltd (Shandong, China). Both the samples were analyzed by fluorescence flow cytometry (DxFLEX, BECKMAN COULTER, USA). Other detection steps were carried out according to the operating instructions on the kit used.

### Sample preparation and Liquid Chromatography-Mass Spectrometry (LC–MS) analysis

Blood samples were centrifuged with 699 g at 4 ℃ for 10 min and 1.5 mL of each supernatant was transferred to fresh Eppendorf tubes to obtain serum samples, which were stored at -80℃ until use.

Serum samples were thawed in the ice water bath and vortexed for 30 s. 50 μL of the sample was added to a fresh Eppendorf tube with 250 μL pure water. Then 1200 µL extraction solution with internal standard (methanol: acetonitrile, v:v = 1:1, internal standard containing isotope, pre-cooled at − 40 °C) were added and vortexed for 30 s. The mixed liquid were kept in the ice water bath for 15 min and then at – 40 °C for 2 h, which were centrifuged with 13,800 g at 4 °C for 15 min and transferred 1000 μL supernatant into a new Eppendorf tube. The supernatant was centrifuged and concentrated until dry and redissolved with 100 µL 60% acetonitrile, swirled for 30 s, kept in the ice water bath for 5 min, and centrifuged (13,800 g) at 4℃for 15 min. The clear supernatants were transferred to LC vials and then stored at 4℃ until analysis.

Samples were analyzed by 600 Multiple Reaction Monitoring (600 MRM), one kind of targeted metabolomics, with LC–MS. ACQUITY UPLC PREMIER (Waters) with a Waters Atlantis Premier BEH Z-HILIC Column (1.7 μm, 2.1 × 150 mm) was used for LC analysis. Mobile phases A and B were ultra-pure water/ acetonitrile (8/2) and cetonitrile/ultra-pure water (9/1), respectively, both containing 10 mmol/L ammonium acetate. SCIEX 6500 QTRAP + (AB Sciex) triple quadrupole mass spectrometer equipped with IonDrive Turbo V ESI ion source was used for MS in MRM mode. Temperature was set at 500 °C. IonSpray Voltage was set as + 5000 V/−4500 V. Curtain gas, ion source gas 1, and ion source gas 2 were set as 35 psi, 50 psi, and 50 psi, respectively.

### Statistical analysis

The categorical variables were described by frequencies and proportions, and the continuous variables were described by medians and interquartile ranges (IQRs). Comparisons between groups were performed by the Chi-square test or Fisher’s exact test. The logistic regression model was used to explore the effects of demographic variables on homoarginine levels. The Spearman rank correlation coefficient with associated p-value was calculated to measure the relationship between variables. All statistical analyses were performed by R version 4.1.2 (R Development Core Team). A two-sided P < 0.05 was considered statistically significant.

## Results

### Demographic characteristics

Totally 46 COVID-19 patients were enrolled, including 18 in mild group and 28 in severe group (19 survival and 9 fatal) (Supplemental Fig. 1). Serum samples, urine samples, and stool samples were collected at the acute stage for all 46 COVID-19 patients and at the recovery stage for 19 survival patients. No significant differences were observed between mild and severe groups or survival and fatal groups in terms of sex, body mass index (BMI), smoking, drinking, underlying diseases and clinical manifestations (Table 1). The patients were older in the fatal group than in the survival group (P < 0.001). The hospital stay of severe patients was significantly longer than that of mild patients (P = 0.001).

**Table 1:** General and clinical characteristics of COVID-19 patients

Variables	Mild (n = 18)	Severe (n = 28)			P^#^
		Survival (n = 19)	Dead (n = 9)	P^*^	
Sex, male, n (%)	16 (88.9)	11 (57.9)	7 (77.8)	0.417	0.090
Age, years, n (%)				** < 0.001**	0.548
< 65	6 (33.3)	5 (26.3)	0 (0)		
65–75	6 (33.3)	11 (57.9)	1 (11.1)		
≥ 75	6 (33.3)	3 (15.8)	8 (88.9)		
BMI, kg/m^2^, mean ± SD	25.6 ± 2.2	25.1 ± 3.5	25.0 ± 2.4	0.928	0.580
Smoking, n (%)	1 (5.6)	2 (10.5)	1 (11.1)	1	1
Drinking, n (%)	1 (5.6)	2 (10.5)	0 (0)	1	1
Underlying disease, n (%)					
Hypertension	12 (66.7)	10 (52.6)	7 (77.8)	0.250	0.761
Diabetes	6 (33.3)	8 (42.1)	4 (44.4)	1	0.554
Coronary heart disease	2(11.1)	5 (26.3)	3 (33.3)	1	0.274
Cerebral infarction	3(16.7)	3 (15.8)	2 (22.2)	1	1
Clinical manifestations					
Fever, n (%)	14 (77.8)	14 (73.7)	6 (66.7)	1	0.740
Highest body temperature, ℃, mean ± SD	38.7 ± 0.7	39.1 ± 0.6	39.0 ± 0.6	0.634	0.120
Cough, n (%)	13 (72.2)	16 (84.2)	8 (88.9)	1	0.284
Expectoration, n (%)	12 (66.7)	14 (73.7)	6 (66.7)	1	0.753
Dyspnea, n (%)	9 (50.0)	13 (68.4)	9 (100)	0.136	0.058
Polypnea, n (%)	2 (11.1)	2 (10.5)	4 (44.4)	0.064	0.453
Wheezing, n (%)	1 (5.6)	2 (10.5)	2 (22.2)	0.574	0.634
Hospital stays, mean ± SD	7 ± 2	13 ± 7	11 ± 8	0.631	**0.001**

The significance of bold means *P* < 0.05

*SD* standard deviation

*Between survival and fatal groups

^#^Between mild and severe groups

### Relationships between homoarginine and arginine or disease severity

**Fig. 1:**
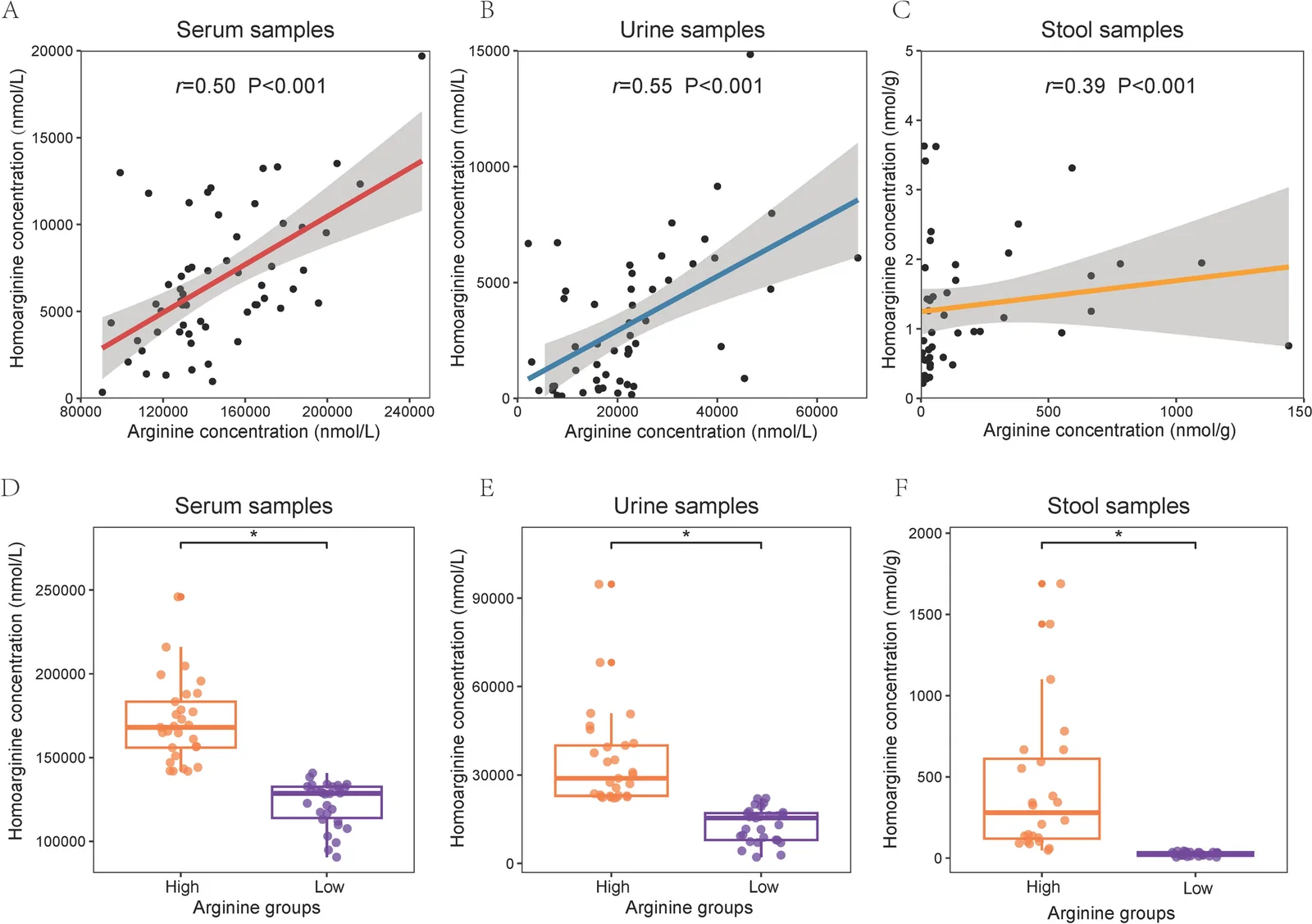
The correlation between the concentrations of homoarginine and arginine. **A** The concentrations of homoarginine and arginine in serum samples. Datapoints are index values. The line is the fitted curve and the shadow is its 95% confidence interval. **B** The concentrations of homoarginine and arginine in urine samples. **C** The concentrations of homoarginine and arginine in stool samples. **D** The concentrations of homoarginine in serum samples of different arginine concentration groups. **E** The concentrations of homoarginine in urine samples of different arginine concentration groups. **F** The concentrations of homoarginine in stool samples of different arginine concentration groups

The concentration of homoarginine was positively correlated with the concentration of arginine in serum samples (*r* = 0.50), urine samples (*r* = 0.55), and stool samples (*r* = 0.39) (all P < 0.001, Fig. 1A–C). Higher concentrations of homoarginine were detected in the arginine group with higher concentrations across all three samples (all P < 0.05, Fig. 1D–F).

**Fig. 2:**
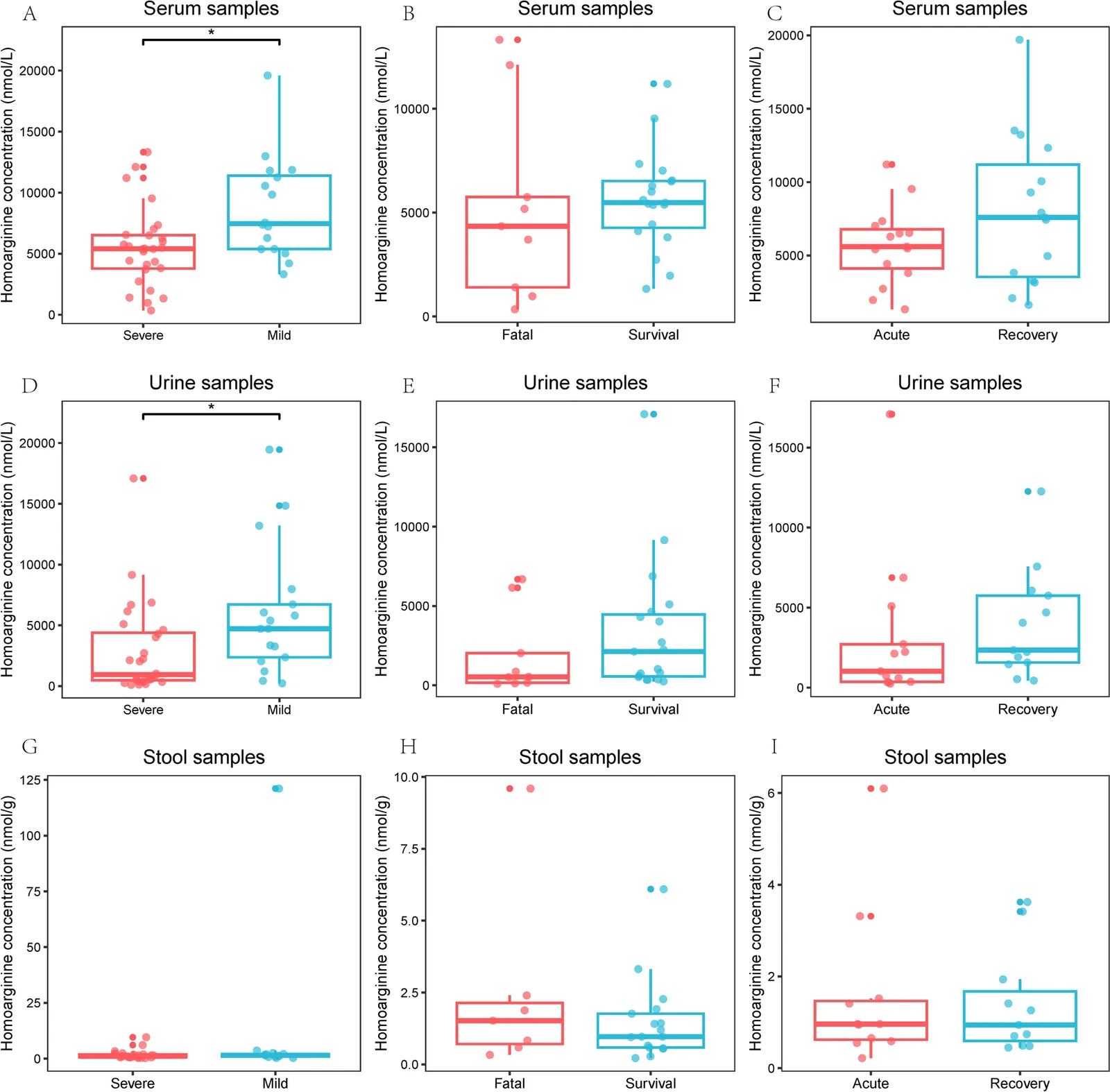
The concentrations of homoarginine in serum, urine, and stool samples of patients with different disease severity. **A** in severe and mild patients from serum; **B** in fatal and survival patients from serum; **C** at acute and recovery stage from serum; **D** in severe and mild patients from urine; **E** in fatal and survival patients from urine; **F** at acute and recovery stage from urine; **G** in severe and mild patients from stool; **H** in fatal and survival patients from stool; **I** at acute and recovery stage from stool

The serum concentration and urine concentration of homoarginine were lower in severe patients than in mild patients (both P < 0.05, Fig. 2). In spite of not being statistically significant, the serum concentration and urine concentration of homoarginine appeared to be lower in fatal patients and at the acute stage. While no differences were observed in stool samples.

**Fig. 3:**
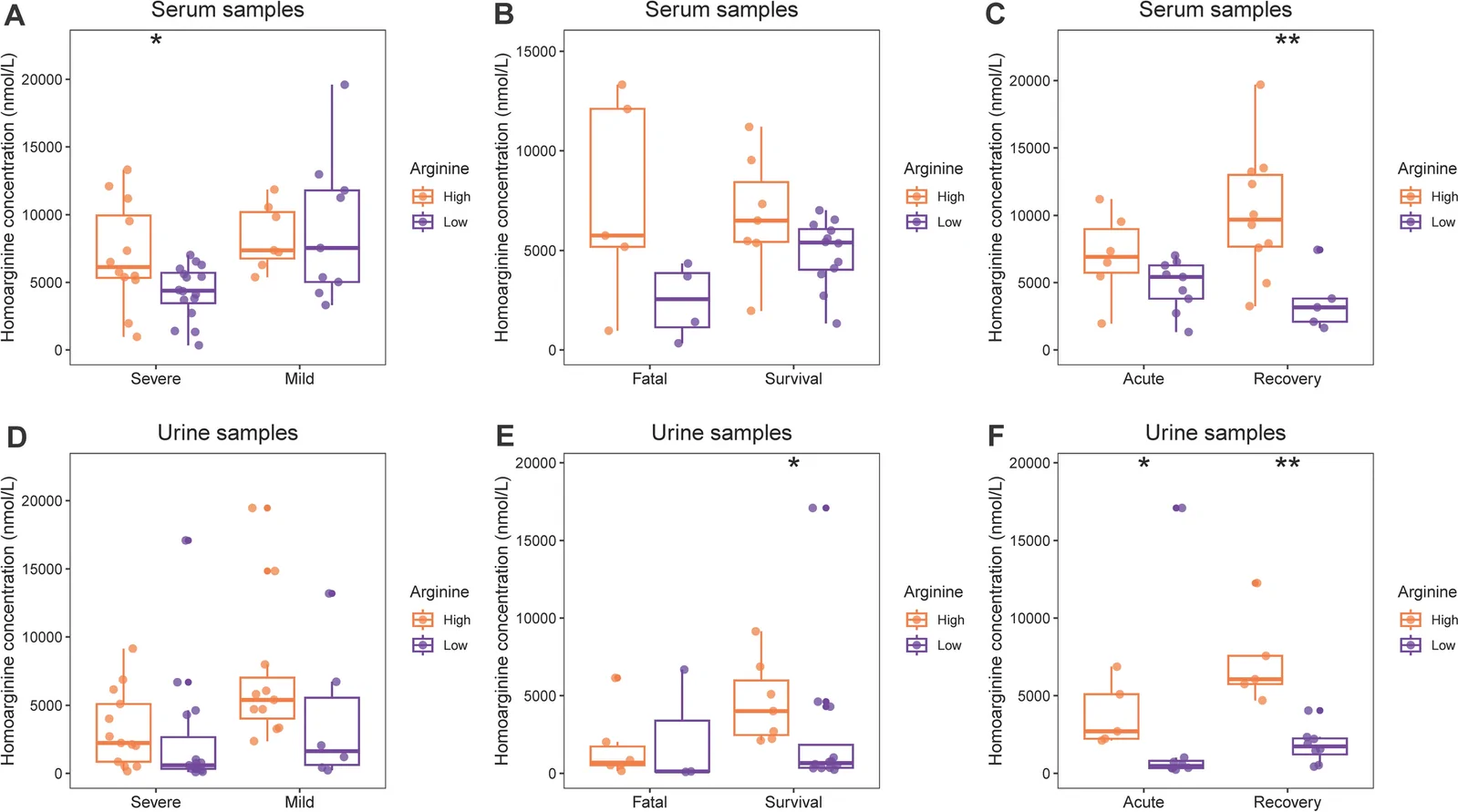
The concentrations of homoarginine in serum and urine samples of different arginine concentration groups and of different disease severity groups. *P < 0.05, **P < 0.01. **A** in severe and mild patients from serum; **B** in fatal and survival patients from serum; **C** at acute and recovery stage from serum; **D** in severe and mild patients from urine; **E** in fatal and survival patients from urine; **F** at acute and recovery stage from urine;

The relationship between the concentrations of homoarginine and arginine varied in different disease severity groups (Fig. 3). For the severe patients, the serum concentration of homoarginine were significantly lower in the patients with a low arginine level than with a high arginine level (P = 0.006), while no significant correlation was detected for mild patients. A big difference of serum homoarginine concentration between the groups of high and low arginine concentrations was also observed for the fatal patients (P = 0.111), different from the survival patients (P = 0.142); however, neither difference reached statistical significance. In contrast, in the urine samples, the difference of serum homoarginine concentration was more pronounced in the mild patients (P = 0.122) and in the survival patients (P = 0.022).

### Relationships between homoarginine and laboratory test results

**Fig. 4:**
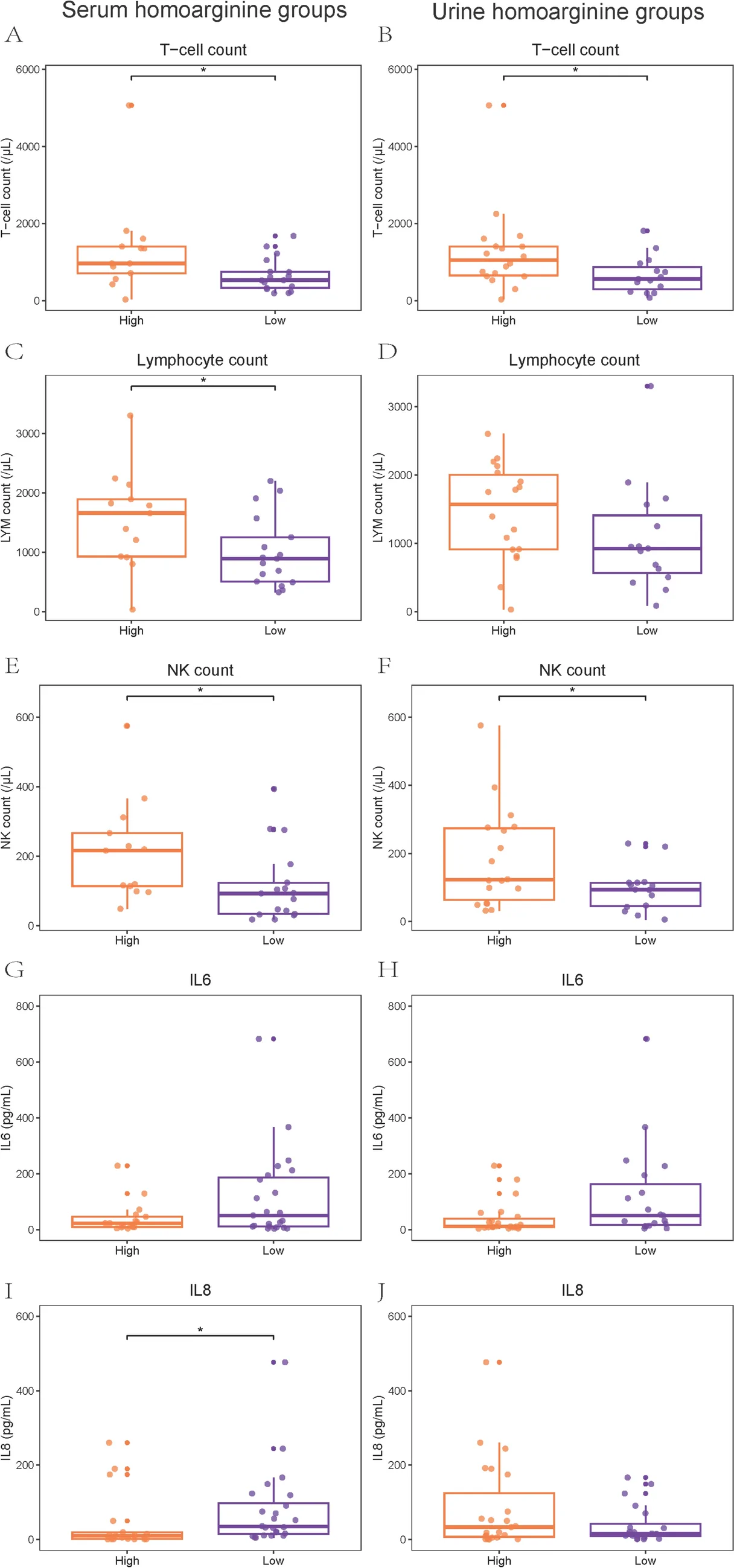
Laboratory test results about immunity function of different serum and urine homoarginine concentration groups and of different disease severity groups. *P < 0.05, **P < 0.01. **A** T-cell count in serum; **B** T-cell count in urine; **C** Lymphocyte count in serum; **D** Lymphocyte count in urine; **E** NK count in serum; **F** NK count in urine; **G** IL-6 in serum; **H** IL-6 in urine; **I** IL-8 in serum; **J** IL-8 in urine

The relationship between disease severity of COVID-19 and 42 immune indexes, including lymphocyte subsets cytokines, and immunoglobulins, was explored (Supplementary Fig. 2). Compared with mild patients, lower percentages of T cell and CD3^+^CD4^+^ T cell, lower counts of CD3^+^CD4^+^ T cell and natural killer (NK) cell, and higher levels of interleukin-6 (IL-6) and granulocyte-colony stimulating factor (GCSF) were observed in severe patients (all P < 0.05). Compared with survival patients, lower counts of T cell, CD3^+^CD4^+^ T cell, and white blood cell (WBC), lower levels of IL-13, CH50, C3, and higher levels of monocyte chemoattractant protein 1 (MCP-1) were observed in patients in fatal group (all P < 0.05). The concentration of IL-8 was significantly lower in patients at the acute stage than at the recovery stage (P = 0.046). Immunology test results of patients with different homoarginine levels were illustrated in Supplementary Fig. 3. The counts of T cell, WBC, and NK cell and the concentrations of IL-8 and IL-9 were related to the level of homoarginine in serum (all P < 0.05). Additionally, there was a negative relationship between the levels of IL-6 and the urine concentration of homoarginine (P = 0.004). Five indicators were statistical significantly correlated with both disease severity and homoarginine concentration, including less counts of T cell, WBC, and NK cell and higher levels of IL-6 and IL-8 in the low concentration of homoarginine group and in severe group or fatal group, or in patients at the acute stage (all P < 0.05, Fig. 4).

**Fig. 5:**
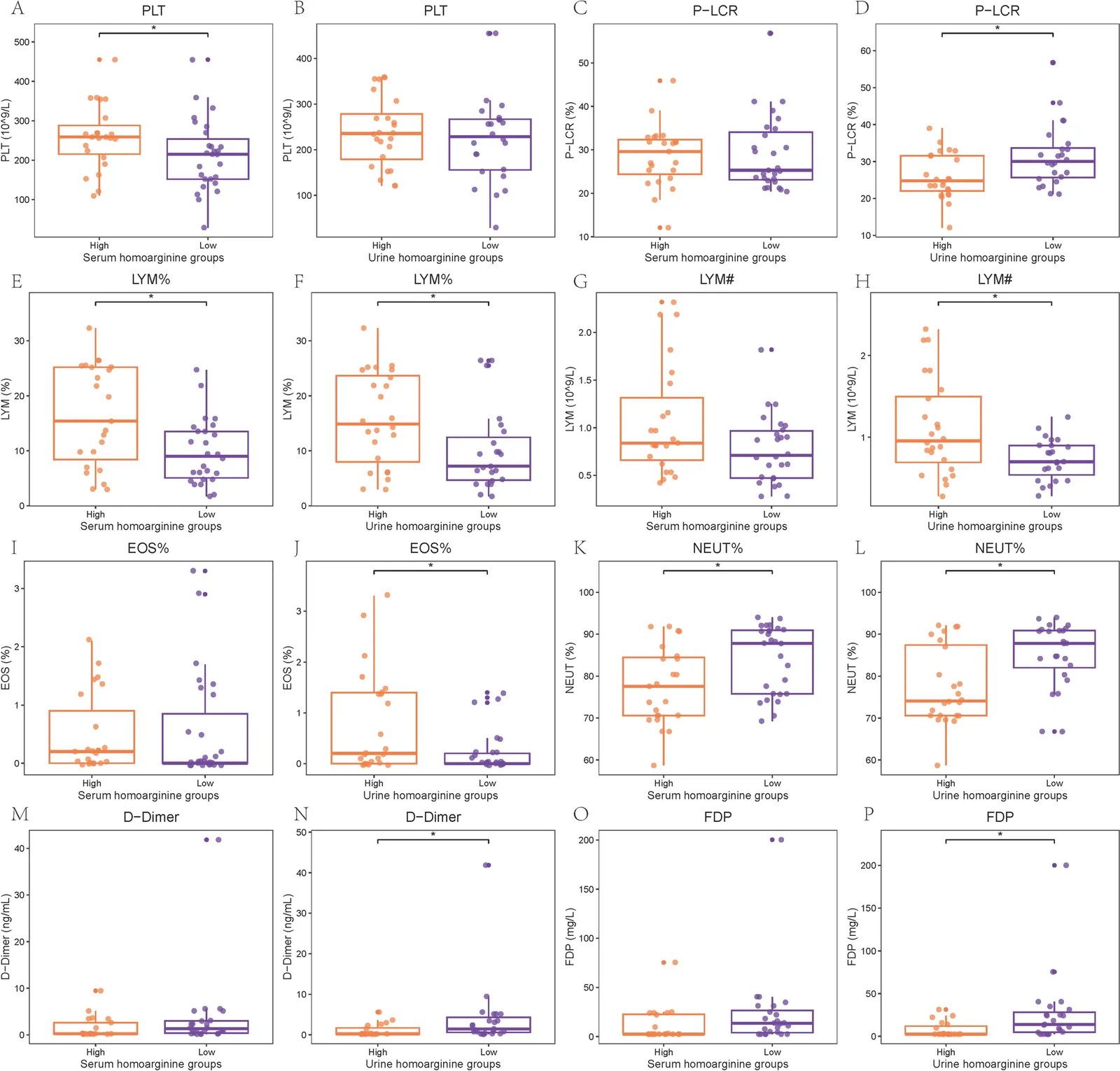
Blood routine test of different serum and urine homoarginine concentration groups and of different disease severity groups. *PLT* platelet, *R-LCR* platelet large cell ratio, *LYM%* percentage of lymphocyte, *LYM#* lymphocyte counts, *EOS%* eosinophil percentage, *NEYT%* neutrophilic granulocyte percentage, *FDP* fibrinogen degradation products

Other laboratory test result of patients in different severity groups, including blood routine test and coagulation function test, were illustrated in Supplementary Fig. 4. Higher WBC counts and neutrophils counts and percentage and lower lymphocyte percentage were observed in patients in the severe and fatal groups compared with that in the mild and survival groups (all P < 0.05). It’s worth noting that over half of the coagulation function test results showed the significant difference between the fatal and survival patients (all P < 0.05). The relationships between the concentration of homoarginine and other laboratory test results were explored (Supplementary Fig. 5). Several test results were statistically associated with the concentration of homoarginine, including but not limited to the counts of platelets, and the percentage of lymphocyte and neutrophils (all P < 0.05). Eight items with the significant association with both disease severity and homoarginine concentration were screened, with six and two items in blood routine test and coagulation function test (all P < 0.05), respectively (Fig. 5). Lower counts of platelets and lymphocyte, lower percentages of lymphocyte, eosinophil, and neutrophilic granulocyte, and higher platelet large cell ratio were observed in patients with low homoarginine levels and in fatal or severe patients (all P < 0.05). In terms of coagulation function, higher levels of D-Dimer and fibrinogen degradation products in patients were associated with low homoarginine concentrations (both P < 0.05) and in fatal group (both P < 0.05).

### Associations between the concentration of homoarginine and demographic characteristics

Patients with hypertension had a significantly lower concentration of serum homoarginine (odds ratio 10.905, 95% confidence interval 1.454 − 137.144, P < 0.001, Table 2). The risk of decreased serum homoarginine was higher in severe patients compared with mild patients (odds ratio 7.695, 95% confidence interval 1.429 − 58.773, P < 0.001). No associations between serum homoarginine levels and sex, age, smoking status, drinking status and underlying diseases except hypertension were observed.

**Table 2:** The associations between the demographic characteristics and lower serum homoarginine levels by the binary logistic regression model

Variables	OR (95%CI)
Sex	
Male	Ref
Female	2.310 (0.304 ~ 22.031)
Age	
< 65	Ref
65–75	0.775 (0.072 ~ 8.794)
≥ 75	5.290 (0.461 ~ 96.071)
Smoking	
No	Ref
Yes	16.452 (0.095 ~ 36,538.040)
Drinking	
No	Ref
Yes	0.304 (0.003 ~ 134.706)
Hypertension	
No	Ref
Yes	**10.905 (1.454 ~ 137.144)**
Diabetes	
No	Ref
Yes	0.159 (0.022 ~ 0.872)
Coronary heart disease	
No	Ref
Yes	3.746 (0.444 ~ 53.734)
Cerebral infarction	
No	Ref
Yes	1.405 (0.107 ~ 16.842)
Severity	
Mild	Ref
Severe	**7.695 (1.429 ~ 58.773)**

The significance of bold means *P* < 0.05

## Discussion

This study revealed a positive association between the concentrations of homoarginine and arginine, suggesting that homoarginine can be generated from arginine by AGAT (Tsikas 2023; Mangoni et al. 2019). In spite of the relatively low concentration of homoarginine in humans, the concentration of homoarginine may have effects on the concentration of arginine as well. Given the similar chemical structure, homoarginine is also a substrate for NOS and arginase like arginine (Atzler et al. 2015), which lead to the hypothesis that the supplementation with homoarginine may be able to reduce the binding of arginine to enzymes thereby reducing the consumption of arginine. The statistically significant relationship between the serum concentration of homoarginine and arginine was shown in severe COVID-19 patients, but not in mild patients. It may due to the increased consumption of arginine in severe patients and the more pronounced interaction between homoarginine and arginine. However, the relationship was not detected in fatal or survival groups, probably because of inadequate sample size.

This study showed lower concentrations of homoarginine in patients in severe or fatal groups compared with that in mild or survival groups, which does not support the previous findings that homoarginine concentration was not associated with disease severity (Haşimi et al. 2023). However, one study observed significantly lower concentration of homoarginine and increased arterial stiffness in post-COVID-19 patients compared with healthy controls, suggesting a potential role for homoarginine in endothelial dysfunction in COVID-19 (Jud et al. 2021). In addition, the correlation between homoarginine concentration and the risk of cardiovascular events has been confirmed by many epidemiological studies (Karetnikova et al. 2019), consistent with the hypothesis that homoarginine affects endothelial function. In this study, patients with hypertension were associated with a significantly higher likelihood of decreased serum concentration of homoarginine, which was in accordance with the correlation between homoarginine levels and the risk of cardiovascular events (Karetnikova et al. 2019).

Although there are few studies on the relationship between homoarginine and COVID-19, it can be hypothesized that homoarginine has a potential effect on COVID-19 through its interference with the arginine pathway (Atzler et al. 2015). In terms of arginine, several metabolomics studies have illustrated that the disturbed arginine metabolism is involved in SARS-CoV-2 infection (Rees et al. 2021; Blasco et al. 2020), with the association between reduced arginine and more severe disease (Rees et al. 2021; Sacchi et al. 2021). This result is open to several possible explanations. A growing body of research suggests endothelial dysfunction as a key role in COVID-19 (Xu et al. 2023; Bonaventura et al. 2021), which is correlated with NO (Cyr et al. 2020) and therefore with arginine as a substrate for NO production by NOS (Khalaf et al. 2019; Gambardella et al. 2020). Given the similar structure, it is also a possible way for homoarginine to affect endothelial function. Immune function is affected by arginine levels (Almeida et al. 2021), including but not limited to diminished T-cell responses in the presence of reduced arginine and increased expression of arginase in cells (Czystowska-Kuzmicz et al. 2019; Lercher et al. 2019; Fletcher et al. 2015) and affected T-cell proliferation due to arginine, possibly through dysregulation of the expression of cyclin D3 and cyclin-dependent kinase 4, which modulate the G1 to S phase transition (Rodriguez et al. 2007). In our study, the counts of T cell were found to positively connected with homoarginine concentration, consistent with the trend of the effect of arginine on T cell. Previous studies have reported that the expression of activated GPIIb/IIIa complexon platelets was negatively correlated with arginine concentration, suggesting the effect of arginine on coagulation function (Sacchi et al. 2021). Consistently, the concentration of homoarginine was found to be negatively connected with the levels of D-Dimer and fibrinogen degradation products, supporting the association of homoarginine and abnormal coagulation status. The administration of arginine has been proved to boost immune system (Tepaske et al. 2001; Popovic et al. 2007) and increase NO bioavailability and hence improve endothelial function (Nagaya et al. 2001; Brown et al. 2018). A randomized, double-bind, placebo-controlled, parallel trial revealed that additional oral arginine supplementation in patients with COVID-19 led to significantly reduced levels of pro-inflammatory IL-2, IL-6, and IFN-γ (Fiorentino et al. 2021). In our study, pro-inflammatory IL-6 and IL-8 were found to negatively connected with homoarginine concentration. In patients with arginine administration, the in-hospital stay was significantly reduced compared with patients taking placebo (Fiorentino et al. 2021). In terms of long COVID-19, the administration of arginine plus vitamin C was shown to restored serum l-arginine concentrations, improved endothelial function and walking performance and relieved symptoms, including dyspnea, asthenia, and chest tightness (Tosato et al. 2022; Izzo et al. 2022).

Considering the effect of homoarginine on body, the effect of arginine on COVID-19, and the interaction between arginine and homoarginine, the administration of homoarginine appears to be meaningful in clinical treatment. The orally administered homoarginine was readily absorbed in the intestine and almost recovered in urine without metabolism in pigs and rats (Stockebrand et al. 2015; Hou et al. 2016). Previous studies have showed that oral daily supplementation with homoarginine can significantly improve the serum concentration of homoarginine in young volunteers, which provide a theoretical basis for homoarginine administration (Atzler et al. 2016). An animal study reported that the supplementation of arginine and homoarginine both showed positive effects on amelioration of oxidative stress (Chetla et al. 2022), suggesting potential therapeutic effects. In reviewing the literature, no data was found on the effect of homoarginine administration on COVID-19, which would be a fruitful area for further work.

There were some limitations in our study. This study was mainly limited by the relatively small sample size. Despite the limitations in sample size, our study broadened the existing knowledge about the relationships between homoarginine concentration, arginine concentration and disease severity in COVID-19 patients. Unfortunately, this study did not include laboratory tests on endothelial functions, leaving the way in which homoarginine affects disease severity unclear.

## Conclusion

The homoarginine concentration was positively correlated with arginine concentration and negatively correlated with disease severity. Homoarginine concentrations were correlated with several indicators of immunity and coagulation. The metabolic mechanism of homoarginine needs to be further investigated to explore its potential role for the treatment of COVID-19.

## Supplementary Information

Below is the link to the electronic supplementary material.

Supplementary file1 Supplemental Figure 1. The classification of COVID-19 patients and the serum, urine, and stool samples collection (TIF 1604 KB)

Supplementary file2 Supplemental Figure 2. 42 laboratory test results about immunity function of different disease severity groups. A-C: percentage of T cell; D-F: percentage of CD3+CD4+ T cell; G-I: percentage of CD3+CD8+ T cell; J-L: percentage of white blood cell; M-O: percentage of natural kill cell; P-R: percentage of CD3+CD56+ T cell; S-U: percentage of B cell; V-X: counts of T cell; Y-AA: counts of CD3+CD4+ T cell; AB-AD: counts of CD3+CD8+ T cell; AE-AG: counts of white blood cell; AH-AJ: counts of natural kill cell; AK-AM: counts of CD3+CD56+ T cell; AN-AP: counts of B cell; AQ-AS: ratio of CD4+ T cell/CD8+ T cell; AT-AV: percentage of regulatory T cells; AW-AY: concentration of IL-10; AZ-BB: concentration of IL12p70; BC-BE: concentration of IL-17; BF-BH: concentration of interferon-α; BI-BK: concentration of interferon-γ; BL-BN: tumor necrosis factor-α; BO-BQ: concentration of IL-1β; BR-BT: concentration of IL-2; BU-BW: concentration of IL-4; BX-BZ: concentration of IL-5; CA-CC: concentration of IL-4; CD-CF: concentration of IL-5; CG-CI: concentration of IL-4; CJ-CL: concentration of IL-5; CM-CO: granulocyto-colony stimulating factor, GCSF; CP-CR: human granulocyte-macrophage colony stimulating factor, GMCSF; CS-CU: vascular endothelial growth factor, VEGF; CV-CX: macrophage inflammatory protein 1 alpha, MIP-1α; CY-DA: active monocyte chemotactic protein 1, MCP-1; DB-DD: 50% hemolytic unit of complement, CH50; DE-DG: immunoglobulin G, IgG; DH-DJ: immunoglobulin A, IgA; DK-DM: immunoglobulin M, IgM; DN-DP: immunoglobulin E, IgE; DQ-DS: complement 3, C3; DT-DV: complement 4, C4 (JPG 2426 KB)

Supplementary file3 Supplemental Figure 3. 42 laboratory test results about immunity function of different serum and urine homoarginine concentration groups A-B: percentage of T cell; C-D: percentage of CD3+CD4+ T cell; E-F: percentage of CD3+CD8+ T cell; G-H: percentage of white blood cell; I-J: percentage of natural kill cell; K-L: percentage of CD3+CD56+ T cell; M-N: percentage of B cell; O-P: counts of T cell; Q-R: counts of CD3+CD4+ T cell; S-T: counts of CD3+CD8+ T cell; U-V: counts of white blood cell; W-X: counts of natural kill cell; Y-Z: counts of CD3+CD56+ T cell; AA-AB: counts of B cell; AC-AD: ratio of CD4+ T cell/CD8+ T cell; AE-AF: percentage of regulatory T cells; AG-AH: concentration of IL-10; AI-AJ: concentration of IL12p70; AK-AL: concentration of IL-17; AM-AN: concentration of interferon-α; AO-AP: concentration of interferon-γ; AQ-AR: tumor necrosis factor-α; AS-AT: concentration of IL-1β; AU-AV: concentration of IL-2; AW-AX: concentration of IL-4; AY-AZ: concentration of IL-5; BA-BB: concentration of IL-4; BC-BD: concentration of IL-5; BE-BF: concentration of IL-4; BG-BH: concentration of IL-5; BI-BJ: granulocyto-colony stimulating factor, GCSF; BK-BL: human granulocyte-macrophage colony stimulating factor, GMCSF; BM-BN: vascular endothelial growth factor, VEGF; BO-BP: macrophage inflammatory protein 1 alpha, MIP-1α; BQ-BR: active monocyte chemotactic protein 1, MCP-1; BS-BT: 50% hemolytic unit of complement, CH50; BU-BV: immunoglobulin G, IgG; BW-BX: immunoglobulin A, IgA; BY-BZ: immunoglobulin M, IgM; CA-CB: immunoglobulin E, IgE; CC-CD: complement 3, C3; CE-CF: complement 4, C4. (JPG 1782 KB)

Supplementary file4 Supplemental Figure 4. 34 laboratory test results about immunity function of different disease severity groups. WBC: white blood cell count; RBC: red blood cell count; MCV: mean corpuscular volume; HGB: hemoglobin; HCT: hematocrit; PLT: platelet; MCH: mean corpuscular hemoglobin; MCHC: mean corpuscular hemoglobin concentration; PCT: plateletcrit; MPV: mean platelet volume; PDW: platelet distribution width; P-LCR: platelet large cell ratio; MON%: monocyte ratio; MON#: monocyte count; LYM%: lymphocyte ratio; LYM#: lymphocyte count; BASO%: basophil ratio; BASO#: basophil count; EOS%: eosinophil ratio; EOS#: eosinophil count; NEUT#: neutrophil count; NEUT%: neutrophil ratio; RDW-CV: coefficient variation of red cell distribution width; RDW-SD: standard deviation in red cell distribution width; PT: prothrombin time; INR: international normalized ratio; FIB: fibrinogen; APTT: activated partial thromboplastin time; TT: thrombin time; PTA: prothrombin time activity percentage; TT-r: thrombin time ratio; APTT-r: activated partial thromboplastin time ratio; FDP: fibrinogen degradation products. (JPG 1204 KB)

Supplementary file5 Supplemental Figure 5. 34 laboratory test results about immunity function of different serum and urine homoarginine concentration groups WBC: white blood cell count; RBC: red blood cell count; MCV: mean corpuscular volume; HGB: hemoglobin; HCT: hematocrit; PLT: platelet; MCH: mean corpuscular hemoglobin; MCHC: mean corpuscular hemoglobin concentration; PCT: plateletcrit; MPV: mean platelet volume; PDW: platelet distribution width; P-LCR: platelet large cell ratio; MON%: monocyte ratio; MON#: monocyte count; LYM%: lymphocyte ratio; LYM#: lymphocyte count; BASO%: basophil ratio; BASO#: basophil count; EOS%: eosinophil ratio; EOS#: eosinophil count; NEUT#: neutrophil count; NEUT%: neutrophil ratio; RDW-CV: coefficient variation of red cell distribution width; RDW-SD: standard deviation in red cell distribution width; PT: prothrombin time; INR: international normalized ratio; FIB: fibrinogen; APTT: activated partial thromboplastin time; TT: thrombin time; PTA: prothrombin time activity percentage; TT-r: thrombin time ratio; APTT-r: activated partial thromboplastin time ratio; FDP: fibrinogen degradation products (JPG 1436 KB)

## Data Availability

No datasets were generated or analysed during the current study.
