# Supplementary figures and images for: Association of homoarginine with arginine and disease severity in COVID-19 patients
Source: Amino Acids. 2025 May 7;57(1):24. doi: 10.1007/s00726-025-03453-6 (PMC12058869; doi:10.1007/s00726-025-03453-6)

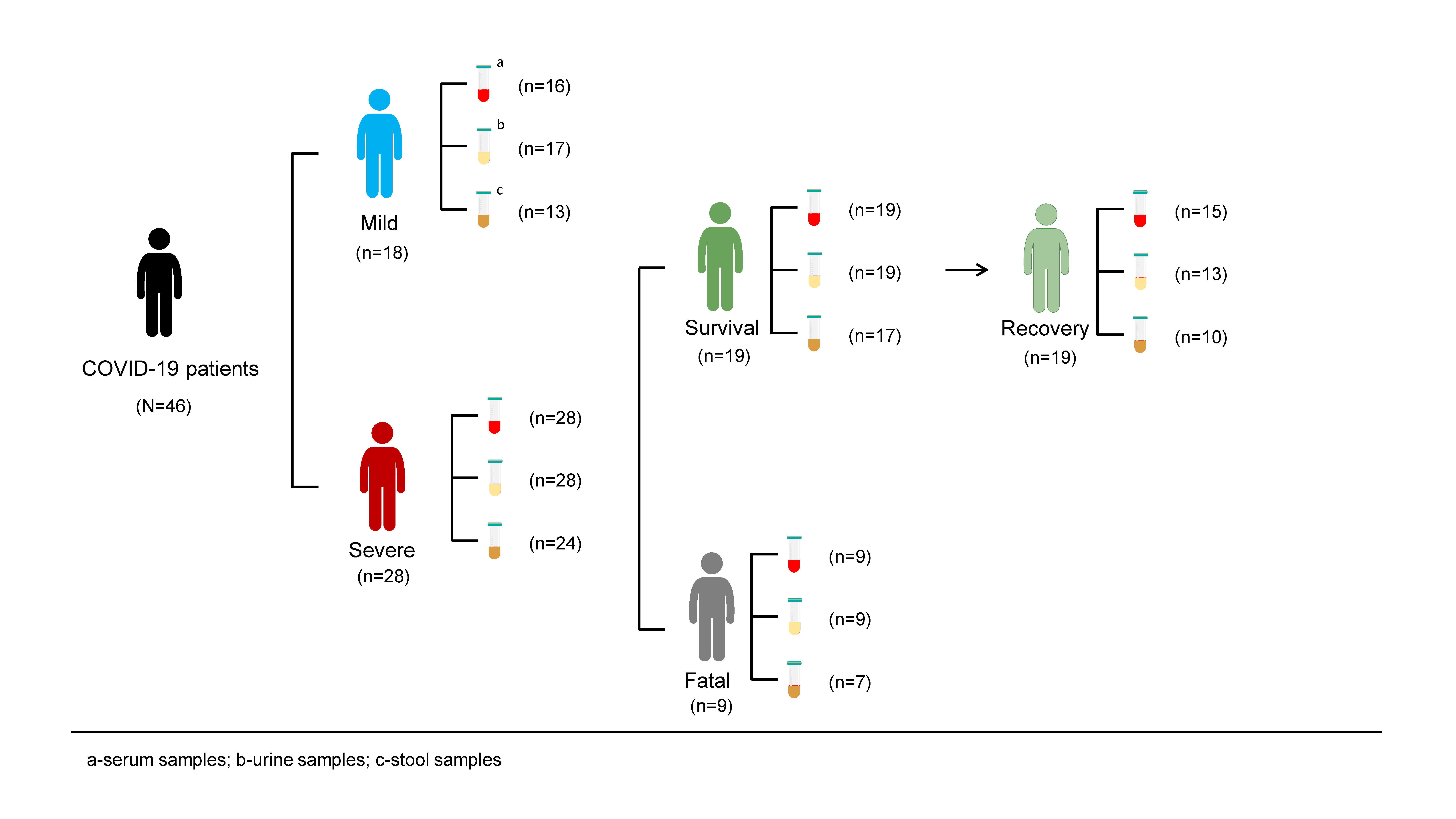

Supplement: Supplementary file 1 — Supplementary file1 Supplemental Figure 1. The classification of COVID-19 patients and the serum, urine, and stool samples collection (TIF 1604 KB) [file 726_2025_3453_MOESM1_ESM.tif]

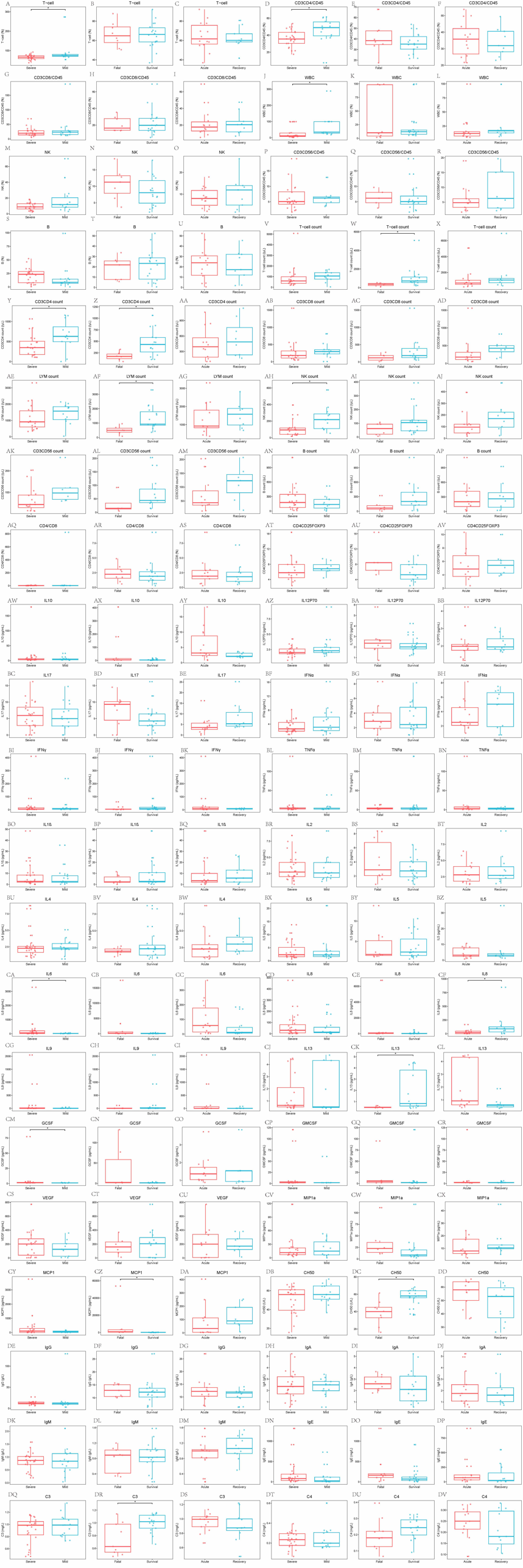

Supplement: Supplementary file 2 — Supplementary file2 Supplemental Figure 2. 42 laboratory test results about immunity function of different disease severity groups. A-C: percentage of T cell; D-F: percentage of CD3+CD4+ T cell; G-I: percentage of CD3+CD8+ T cell; J-L: percentage of white blood cell; M-O: percentage of natural kill cell; P-R: percentage of CD3+CD56+ T cell; S-U: percentage of B cell; V-X: counts of T cell; Y-AA: counts of CD3+CD4+ T cell; AB-AD: counts of CD3+CD8+ T cell; AE-AG: counts of white blood cell; AH-AJ: counts of natural kill cell; AK-AM: counts of CD3+CD56+ T cell; AN-AP: counts of B cell; AQ-AS: ratio of CD4+ T cell/CD8+ T cell; AT-AV: percentage of regulatory T cells; AW-AY: concentration of IL-10; AZ-BB: concentration of IL12p70; BC-BE: concentration of IL-17; BF-BH: concentration of interferon-α; BI-BK: concentration of interferon-γ; BL-BN: tumor necrosis factor-α; BO-BQ: concentration of IL-1β; BR-BT: concentration of IL-2; BU-BW: concentration of IL-4; BX-BZ: concentration of IL-5; CA-CC: concentration of IL-4; CD-CF: concentration of IL-5; CG-CI: concentration of IL-4; CJ-CL: concentration of IL-5; CM-CO: granulocyto-colony stimulating factor, GCSF; CP-CR: human granulocyte-macrophage colony stimulating factor, GMCSF; CS-CU: vascular endothelial growth factor, VEGF; CV-CX: macrophage inflammatory protein 1 alpha, MIP-1α; CY-DA: active monocyte chemotactic protein 1, MCP-1; DB-DD: 50% hemolytic unit of complement, CH50; DE-DG: immunoglobulin G, IgG; DH-DJ: immunoglobulin A, IgA; DK-DM: immunoglobulin M, IgM; DN-DP: immunoglobulin E, IgE; DQ-DS: complement 3, C3; DT-DV: complement 4, C4 (JPG 2426 KB) [file 726_2025_3453_MOESM2_ESM.jpg]

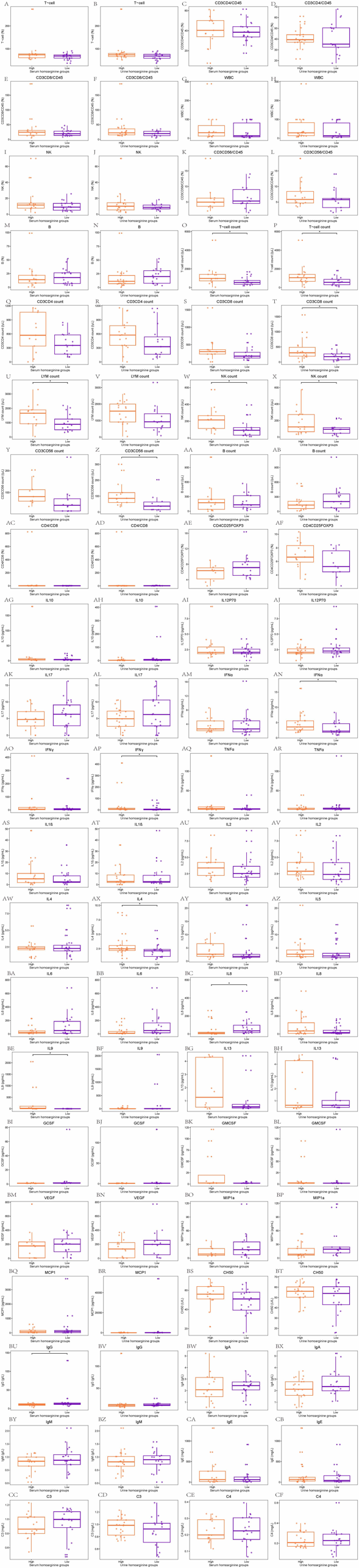

Supplement: Supplementary file 3 — Supplementary file3 Supplemental Figure 3. 42 laboratory test results about immunity function of different serum and urine homoarginine concentration groups A-B: percentage of T cell; C-D: percentage of CD3+CD4+ T cell; E-F: percentage of CD3+CD8+ T cell; G-H: percentage of white blood cell; I-J: percentage of natural kill cell; K-L: percentage of CD3+CD56+ T cell; M-N: percentage of B cell; O-P: counts of T cell; Q-R: counts of CD3+CD4+ T cell; S-T: counts of CD3+CD8+ T cell; U-V: counts of white blood cell; W-X: counts of natural kill cell; Y-Z: counts of CD3+CD56+ T cell; AA-AB: counts of B cell; AC-AD: ratio of CD4+ T cell/CD8+ T cell; AE-AF: percentage of regulatory T cells; AG-AH: concentration of IL-10; AI-AJ: concentration of IL12p70; AK-AL: concentration of IL-17; AM-AN: concentration of interferon-α; AO-AP: concentration of interferon-γ; AQ-AR: tumor necrosis factor-α; AS-AT: concentration of IL-1β; AU-AV: concentration of IL-2; AW-AX: concentration of IL-4; AY-AZ: concentration of IL-5; BA-BB: concentration of IL-4; BC-BD: concentration of IL-5; BE-BF: concentration of IL-4; BG-BH: concentration of IL-5; BI-BJ: granulocyto-colony stimulating factor, GCSF; BK-BL: human granulocyte-macrophage colony stimulating factor, GMCSF; BM-BN: vascular endothelial growth factor, VEGF; BO-BP: macrophage inflammatory protein 1 alpha, MIP-1α; BQ-BR: active monocyte chemotactic protein 1, MCP-1; BS-BT: 50% hemolytic unit of complement, CH50; BU-BV: immunoglobulin G, IgG; BW-BX: immunoglobulin A, IgA; BY-BZ: immunoglobulin M, IgM; CA-CB: immunoglobulin E, IgE; CC-CD: complement 3, C3; CE-CF: complement 4, C4. (JPG 1782 KB) [file 726_2025_3453_MOESM3_ESM.jpg]

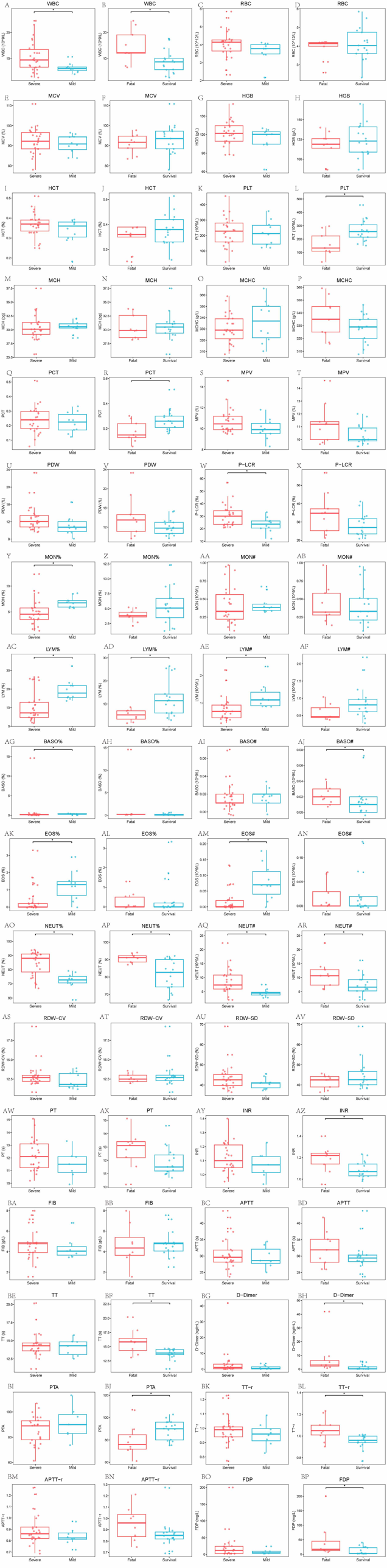

Supplement: Supplementary file 4 — Supplementary file4 Supplemental Figure 4. 34 laboratory test results about immunity function of different disease severity groups. WBC: white blood cell count; RBC: red blood cell count; MCV: mean corpuscular volume; HGB: hemoglobin; HCT: hematocrit; PLT: platelet; MCH: mean corpuscular hemoglobin; MCHC: mean corpuscular hemoglobin concentration; PCT: plateletcrit; MPV: mean platelet volume; PDW: platelet distribution width; P-LCR: platelet large cell ratio; MON%: monocyte ratio; MON#: monocyte count; LYM%: lymphocyte ratio; LYM#: lymphocyte count; BASO%: basophil ratio; BASO#: basophil count; EOS%: eosinophil ratio; EOS#: eosinophil count; NEUT#: neutrophil count; NEUT%: neutrophil ratio; RDW-CV: coefficient variation of red cell distribution width; RDW-SD: standard deviation in red cell distribution width; PT: prothrombin time; INR: international normalized ratio; FIB: fibrinogen; APTT: activated partial thromboplastin time; TT: thrombin time; PTA: prothrombin time activity percentage; TT-r: thrombin time ratio; APTT-r: activated partial thromboplastin time ratio; FDP: fibrinogen degradation products. (JPG 1204 KB) [file 726_2025_3453_MOESM4_ESM.jpg]

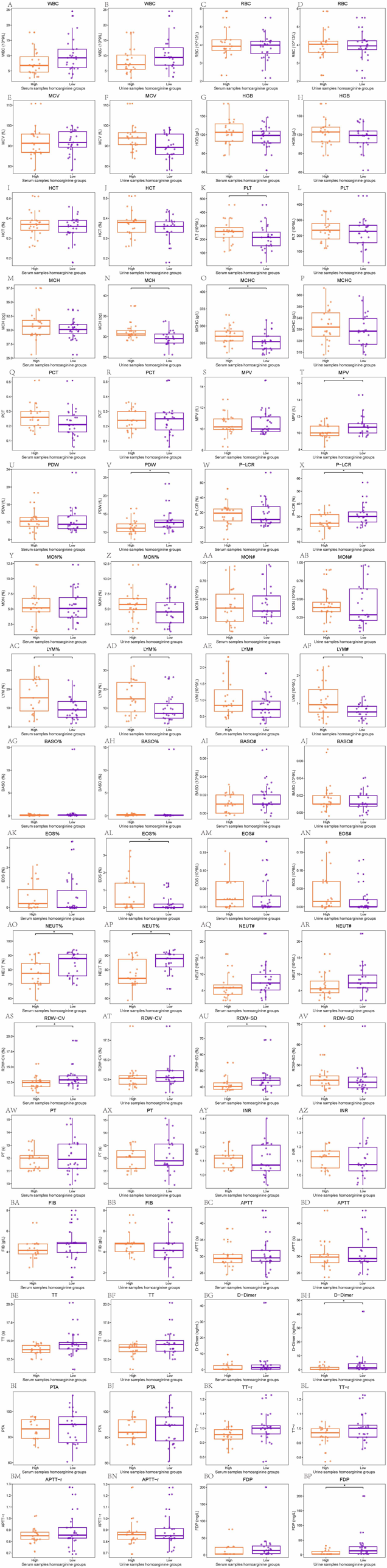

Supplement: Supplementary file 5 — Supplementary file5 Supplemental Figure 5. 34 laboratory test results about immunity function of different serum and urine homoarginine concentration groups WBC: white blood cell count; RBC: red blood cell count; MCV: mean corpuscular volume; HGB: hemoglobin; HCT: hematocrit; PLT: platelet; MCH: mean corpuscular hemoglobin; MCHC: mean corpuscular hemoglobin concentration; PCT: plateletcrit; MPV: mean platelet volume; PDW: platelet distribution width; P-LCR: platelet large cell ratio; MON%: monocyte ratio; MON#: monocyte count; LYM%: lymphocyte ratio; LYM#: lymphocyte count; BASO%: basophil ratio; BASO#: basophil count; EOS%: eosinophil ratio; EOS#: eosinophil count; NEUT#: neutrophil count; NEUT%: neutrophil ratio; RDW-CV: coefficient variation of red cell distribution width; RDW-SD: standard deviation in red cell distribution width; PT: prothrombin time; INR: international normalized ratio; FIB: fibrinogen; APTT: activated partial thromboplastin time; TT: thrombin time; PTA: prothrombin time activity percentage; TT-r: thrombin time ratio; APTT-r: activated partial thromboplastin time ratio; FDP: fibrinogen degradation products (JPG 1436 KB) [file 726_2025_3453_MOESM5_ESM.jpg]
